# Unresectable ectopic hepatocellular carcinoma in the left sub-diaphragmatic area and retroperitoneum treated with TACE: case report and literature review

**DOI:** 10.1080/15384047.2025.2510029

**Published:** 2025-06-21

**Authors:** Chao-Bang Xie, Zi-Jian Tang, Yang Wu, Kai-Fei Zhao

**Affiliations:** Department of Radiology, Affiliated Hospital of Zunyi Medical University, Zunyi, Guizhou Province, China

**Keywords:** Ectopic liver, ectopic hepatocellular carcinoma, hepatocellular carcinoma, metastasis, transarterial chemoembolization, case report

## Abstract

Ectopic hepatocellular carcinoma (EHCC) demonstrates morphological and immunohistochemical features identical to conventional intrahepatic hepatocellular carcinoma (HCC), originating from ectopic liver tissue (EL), which is defined as a hepatic organ or tissue not connected to the mother liver. EHCC is a rare disease and difficult to diagnose preoperatively. Therefore, its epidemiology, treatment, and prognosis are not fully elucidated. Herein, we present a case report of a 52-year-old male diagnosed with unresectable EHCC who underwent transarterial chemoembolization (TACE) and succumbed to the disease 13 months after treatment, accompanied by a literature review summarizing the epidemiology while analyzing therapeutic strategies and prognostic outcomes in EHCC through synthesis of published case evidence.

## Introduction

Extrahepatic tissue that communicates with the biliary system is defined as the accessory liver, and that which does not communicate with the biliary system is termed ectopic liver (EL).^1,2^ Typically, EL is recognized within the gallbladder, spleen, pancreas, adrenal gland, Arantius ligament, diaphragm, thorax, retroperitoneum, and omentum.^3^ Compared to the normal liver, EL has an increased propensity for malignancy due to the lack of native vasculature and inability of bile excursion^2^; it has been suggested that the occurrence of EHCC is associated with insufficient biliary drainage and/or decreased blood supply in the EL.^4^ Moreover, the risk factors for HCC arising in the mother liver, such as cirrhosis, viral hepatitis, or other known risk factors, are less relevant to EHCC.^2,5^ However, the specific pathogenesis is yet unknown and needs to be evaluated in future studies.

While HCC represents the most prevalent primary hepatic malignancy and ranks as the fifth leading cause of cancer-related deaths globally,^6^ its development within EL -functionally autonomous liver tissue anatomically separate from the mother organ – constitutes an exceptionally rare clinicopathological entity.^3^ Hitherto, only a few studies have reported the disease. Herein, we reported a case of unresectable EHCC, treated with transarterial chemoembolization (TACE), and conducted a literature review to describe the epidemiology and explore the treatment and prognosis of the disease based on the previously reported cases.

## Case presentation

A 52-year-old male was admitted for abdominal pain to Quanzhou People’s Hospital of Fujian Province (China) 3 months ago. Abdominal contrast-enhanced computed tomography (CECT) was performed, and the radiological diagnosis was an intraperitoneal and retroperitoneal malignant tumor. Because the patient was diagnosed with a malignant tumor, he believed that the treatment was worthless, so he chose to be discharged instead of further treatment. However, during the three months after discharge, the patient experienced frequent and severe pain in the upper left abdominal quadrant and is now admitted to our hospital for abdominal pain. He has been drinking 250 mL 53° liquor for about 30 years every night and smoking 40 cigarettes for approximately 20 years every day. He had no specific family history. However, HBV infection was detected at the time of admission, and antiviral treatment with Entecavir capsules (orally, 0.5 mg once a day) was administered. Physical examination presented normal vital signs, only tenderness and rebound pain in the upper left abdominal quadrant. Laboratory tests: HBV surface antigen (HBsAg), 159.860 (reference: <0.05) IU/mL; HBV surface antibody (HBsAb), 0.350(reference: <10) mIU/mL; HBVe antigen (HBeAg), 0.362(reference: <1) COI; HBV e antibody (HBeAb), 0.01 (reference: <1) COI; HBV core antibody (HBcAb), 10.06 (reference: <1) COI; HBV DNA, 6.512 × 10^2^ (reference: <3 × 10) IU/mL; hepatitis C virus antibody (HCV Ab), negative. Tumor-related cell markers were as follows: alpha-fetoprotein (AFP), 39.2 (reference: <9) ng/mL; ferritin, 679 (reference: 23.9–336.2) μg/L; carbohydrate antigen 19–9 (CA19–9), carbohydrate antigen 72–4 (CA72–4) and carcinoembryonic antigen (CEA), within normal range (Table 1).

**Table 1.** Laboratory tests and tumor marker.

Laboratory tests			Tumor marker		
Variables (Unit)	Result	Reference range	Variables (Unit)	Result	Reference range
HBsAg (IU/mL)	159.860	<0.05	AFP (ng/mL)	39.2	<9
HBsAb(mIU/mL)	0.350	<10	Ferritin (μg/L)	679	23.9~336.2
HBeAg (COI)	0.362	<1	CA19–9 (U/mL)	16	<25
HBeAb (COI)	0.01	<1	CA72–4 (U/mL)	0.8	<6.9
HBcAb (COI)	10.06	<1	CEA (μg/L)	3.4	<5
HBV DNA(IU/mL)	6.512×10^2^	<3×10^1^			
HCV Ab	negative	–			

**Figure 1.**
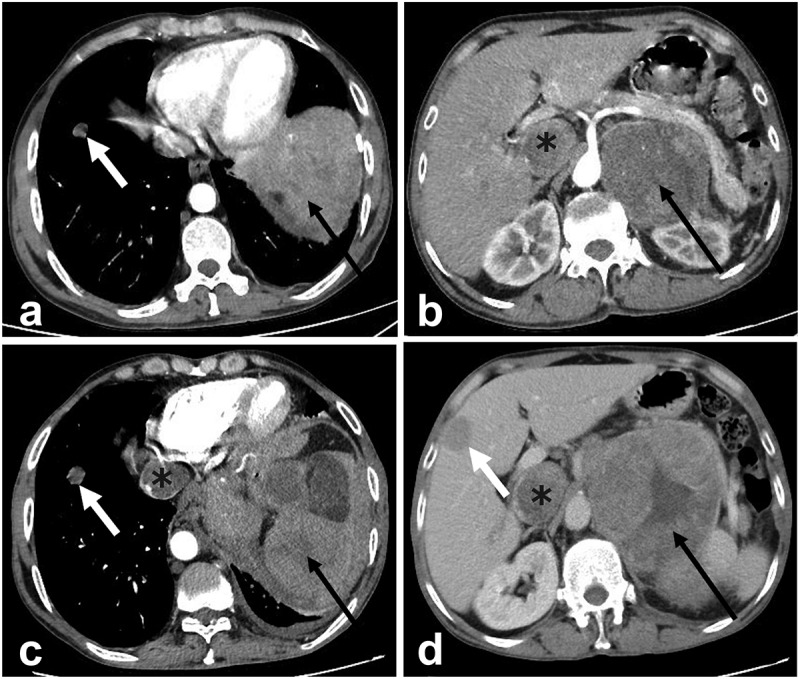
CECT images. (a–d): CECT of the abdomen shows an irregular soft tissue mass in the left sub-diaphragmatic area (a, c: black arrows) and retroperitoneum (b, d: black arrows) with multiple tumor thrombus formations in the IVC (b, c, d: asterisks) and multiple metastases in the lungs (a, c: white arrows) and liver (d: white arrows). A and B are 3 months before and C and D are 3 months after the comparison, and intrahepatic metastases were newly added, and other masses were significantly enlarged. CECT, contrast-enhanced computed tomography; IVC: inferior vena cava.

**Figure 2.**
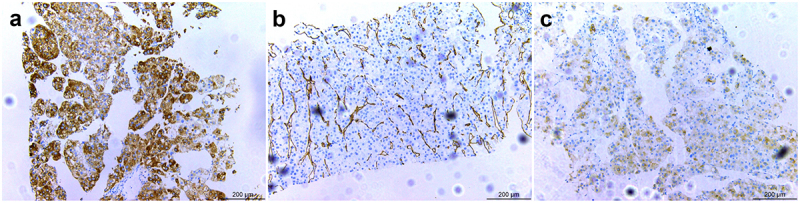
IHC. (a–c): IHC shows that the tumor cells were positive for hep per-1 (a), CD34 demonstrates hepatic sinusoidal vascularization (b) and GPC-3 (c). IHC, immunohistochemistry.

Abdominal CECT, on October 19, 2021, at Quanzhou People’s Hospital revealed an irregular soft tissue mass in the left sub-diaphragmatic area (Figure 1a black arrows) and retroperitoneal area (Figure 1b black arrows), with a tumor size 8.5 × 9.3 × 7.8 cm^3^ in the sub-diaphragm area and 8.3 × 7.5 × 12.1 cm^3^ in the retroperitoneal space; the lesion was enhanced inhomogeneously. Inferior vena cava (IVC) was significantly widened, and tumor thrombus showed heterogeneous enhancement (Figure 1b asterisks). Moreover, no obvious abnormality was observed in the liver. CECT was repeated in our hospital (Affiliated Hospital of Zunyi Medical University, Guizhou Province, China) on January 10, 2022, and sub-diaphragm lesions (Figure 1c black arrows) of 11.2 × 11.5 × 9.8 cm^3^ and retroperitoneal lesions (Figure 1d black arrows) of 11.3 × 9.5 × 14.5 cm^3^ were detected. Also, multiple heterogeneously enhanced tumor thrombi were detected in the IVC (Figure 1c,d asterisks). In addition, multiple round-like hyperdense lesions were observed with slight enhancement in both lungs (Figure 1a,c white arrows) and numerous hypodense lesions in the liver showed slight annular enhancement (Figure 1d white arrows) without suggesting HCC. Numerous lymph nodes were detected in the mediastinum and bilateral lung hilum, retroperitoneal space was enlarged, with a diameter of 0.5–1.5 cm, and mild enhancement was observed. Compared to the imaging examination in other hospitals before 3 months, the left sub-diaphragmatic and retroperitoneal masses were distinctly enlarged. The bilateral lung metastases increased and enlarged, and intrahepatic metastases were newly added. The radiological diagnosis was a malignant tumor in the left abdominal cavity and retroperitoneum with multiple tumor thrombus formations in the IVC and metastases in the liver and lungs. To determine the nature and origin of the lesion, a CT-guided needle biopsy of the abdominal lesion was performed on January 13, 2022. The pathological diagnosis of the biopsy was HCC (Class II) after 3 days. Immunohistochemistry (IHC) showed CK (+), Hep Per-1 (+), Arginase-1(-), AFP (-), GPC-3(+), CK7 (-), CR (-), a-inhibin (-), CgA (-), Syn (±), and CD34-mediated hepatic sinusoidal vascularization (Figure 2). The IHC of biopsy specimens showed that the tumor cells were positive for Hep Per-1 (Figure 2a), and CD34 was the marker for hepatic sinusoidal vascularization (Figure 2b) and GPC-3 (Figure 2c), suggesting the hepatic organ or tissue origin of the carcinoma. However, the liver was normal on CECT 3 months ago, and new slight annular enhancement lesions without suggesting HCC was found in the liver 3 months later. Currently, no link has been established between the tumor in the left sub-diaphragmatic area and retroperitoneum and the liver on CT. Finally, a diagnosis of EHCC in the left sub-diaphragmatic area and retroperitoneum with tumor thrombosis in the IVC and metastasis in the liver and lungs was based on the radiographic and pathological characteristics.

**Figure 3.**
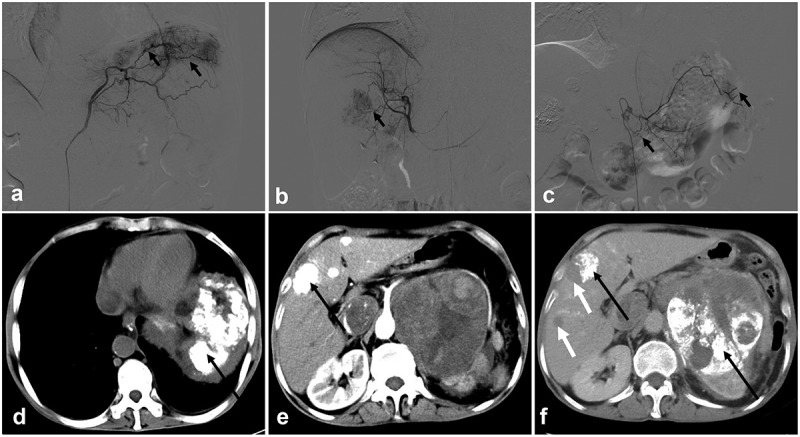
DSA and CECT images. (a–c): DSA of abdominal vessels shows the left inferior phrenic artery (a: black arrows), branches of the left adrenal artery (c: black arrows) and the proper hepatic artery (b: black arrows) were thickened, seem to be tortuous, and are involved in the blood supply of the left sub-diaphragmatic, retroperitoneal, and intrahepatic tumors, respectively. d, e, f: CECT shows high-density shadow deposition in the left sub-diaphragmatic (d: black arrows), retroperitoneal (fig. f black arrows), and intrahepatic (fig. e black arrows) lesions after TACE and increased and enlarged intrahepatic metastases (f white arrows). CECT, contrast-enhanced computed tomography; DSA, digital subtraction angiography; TACE: transarterial chemoembolization.

**Figure 4.**
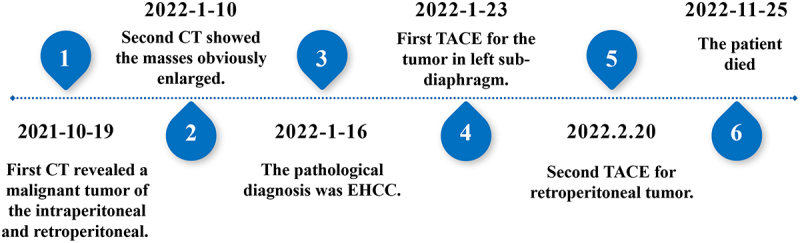
Timeline.

Subsequently, the patient underwent TACE On January 23, 2022. During the operation, digital subtraction angiography (DSA) of abdominal vessels demonstrated that the left inferior phrenic artery was thickened and tortuous, which was involved in the blood supply of the left sub-diaphragmatic tumors (Figure 3a arrows). Moreover, multiple tumors were stained under the left diaphragm. Some branches of the left adrenal artery and left renal artery were thickened and tortuous, participating in the blood supply of the retroperitoneal tumor (Figure 3c arrows). The proper hepatic artery branches were thickened and tortuous, consistent with the DSA manifestations of metastases (Figure 3b arrows). The left sub-diaphragmatic, retroperitoneal, and intrahepatic tumors indicated the need for TACE. Since the patient had several large lesions, simultaneously performing intraperitoneal and retroperitoneal tumor embolization may pose risks, such as Shanghai Xudong Haipu Pharmaceutical Co., LTD. China) +30 mg epirubicin suspension (Shandong New Times Pharmaceutical Co., LTD. China) was selected for embolization of the left sub-diaphragmatic tumor and intrahepatic metastases. After TACE, the liver was protected using compounds, such as glycyrrhizic acid monoamine S, antacids (rabeprazole), and antiemetics (tropisetron hydrochloride injection). One week after the operation, the patient’s abdominal pain symptoms were relieved, and he was discharged. The abdominal CECT on February 18, 2022, showed a high-density shadow in the left sub-diaphragmatic area (Figure 3d black arrows) and intrahepatic region (Figure 3e black arrows), but no abnormal enhancement was found in these lesions. The retroperitoneal mass did not become larger than before 3 months. On February 20, 2022, he was re-admitted to the hospital for TACE treatment for retroperitoneal lesions. On March 30, 2022, CT reexamination showed a high-density shadow in the left sub-diaphragmatic, retroperitoneal, and intrahepatic lesions. The intrahepatic metastases were newly increased (Figure 3f white arrows), while the retroperitoneal tumors were increased slightly. This suggests no obvious progression radiologically 2 months after TACE. The patient was discharged in good condition, including reduced abdominal pain and no complications such as liver or kidney failure or myelosuppression after TACE. At 13 months of follow-up, the patient died. The timeline can be seen in Figure 4.

## Discussion

In the present case, except for HBV, no clinical, radiological, or laboratory indications of primary HCC were detected. Furthermore, previously reported cases of EHCC had a small mass, but our patient had extensive lesions. However, our case involved both the abdominal cavity and the retroperitoneum, which greatly hindered the preoperative diagnosis. Nonetheless, the current report represents the first case involving the abdominal cavity and retroperitoneum.

**Figure 5.**
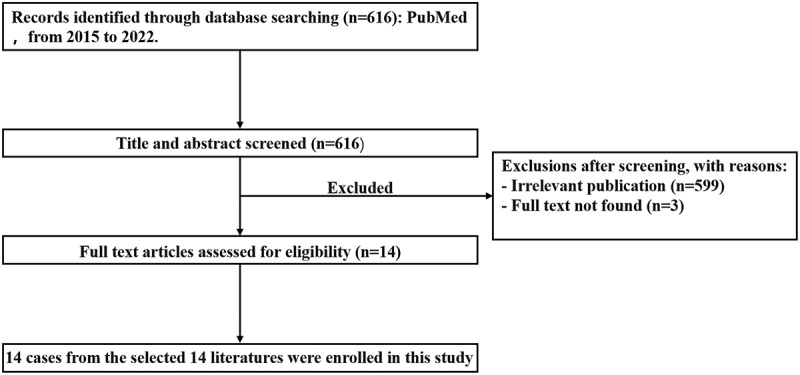
Flowchart of the literature screening process for EHCC. EHCC: ectopic hepatocellular carcinoma.

For the literature review, 616 relevant articles published in English between 2015 and March 31, 2022 were retrieved from PubMed using the keywords “Ectopic hepatocellular carcinoma”. The flowchart of the literature screening process is illustrated in Figure 5. A total of 14 cases were included in this analysis. The following data were collected: the name of the first author, year of publication, patient’s age, sex, location, risk factors, AFP, management, and follow-up results (Table 2).

**Table 2.** Summary of reported cases of EHCC.

Author, Year	Age (years)	Sex	Location	Risk factors	AFP (ng/mL)	Diagnostic methods	Treatment	Outcome
Aaras et al., 2015	64	F	Peritoneum	HBV(+), HCV(-)	200	Laparoscopic resection	Resection	Alive at 4 years
Lee et al., 2015	65	M	Peritoneum	HBV(-), HCV(-)	ND	Surgical resection	Resection	Alive at 17 months
Cui et al., 2016	63	M	Intrathoracic Intraperitoneal	HBV(+)	24793	Surgical resection	Resection Sorafenib	Alive at 13 months
Cheng et al., 2017	54	M	Bile Duct	HBV (+)	3724.75	Surgical resection	Resection	Alive at 3 months
Li et al., 2017	44	F	Pancreas	HBV(-), HCV(-)	>1200	Surgical resection	Resection	Alive at 21 months
George et al., 2017	69	M	Choledochal	HBV (-)	2.9	Biopsy	Resection	ND
Allencherril et al., 2017	82	M	Chest Wall	HBV(-), HCV(-)	ND	Biopsy	No further treatment	Dead at 2 weeks
Jin et al., 2017	56	M	Peritoneum	HBV(-), HCV(-)	8.03	Surgical resection	Resection	Dead at 22 months
Braun et al., 2017	77	M	Pancreas	HBV(-), HCV(-)	Normal	Surgical resection	Resection	Alive at 2 years
Ko et al., 2020	73	M	Peritoneum	HBV(-), HCV(-)	>1000	Laparoscopic resection	Sorafenib	Dead at 1 year
Adachi et al., 2020	81	F	Retroperitoneal	HCV (+)	30.1	Surgical resection	Resection	Alive at 8 months
Rorris et al., 2020	53	M	Adrenal	HCV (+)	Normal	Surgical resection	Resection	ND
Vining et al., 2020	67	M	Pancreas	HBV(-), HCV(-)	Normal	Surgical resection	Resection	ND
Wei et al., 2021	71	M	Adrenal	HBV(-), HCV(-)	1.87	Surgical resection	Resection Lenvatinib	Alive at 10 months
Present case	52	M	Peritoneum Retroperitoneal	HBV(+)	39.2	Biopsy	TACE	Dead at 13 months

EHCC, Ectopic hepatocellular carcinoma; AFP, Alpha-fetoprotein; F, Female; M, Male; HBV, Hepatitis B virus; HCV, Hepatitis C virus; TACE: Transarterial chemoembolization.

In the current study, we included 11 males and 3 females with an average age of 65.6 (range: 44–82) years. This phenomenon suggested that EHCC occurs in elderly patients similar to the HCC of the liver. However, additional EHCC cases are required to elucidate the correlation between the incidence of EHCC and gender. In these cases, the sites of EHCC include the peritoneum, retroperitoneal, intraperitoneal, adrenal, pancreas, bile duct, intrathoracic, and chest wall, similar to those reported previously.^3^ Among these cases, only 5 patients had a history of hepatitis, of which 3 had a hepatitis B virus infection^7–9^ and 2 had a hepatitis C virus disease.^3,10^ This finding indicated that the risk factors for HCC, such as hepatitis B virus or hepatitis C virus infection, alcohol abuse, and cirrhosis, arising in the mother liver are less relevant to EHCC.^5^ Of these 14 cases, only 4 had significantly elevated AFP levels.^5,8,9,11^ AFP is a tumor marker for diagnosing HCC and has prognostic significance. Serum AFP was significantly elevated in about 60% of HCC cases.^12^ Li et al.^11^ speculated that AFP should also be used as a critical marker of EHCC. However, in the current study, the elevated serum AFP may be less frequent than primary HCC. This further increases the difficulty of preoperative diagnosis of EHCC, necessitating additional cases for substantiation of the findings.

Among the cases reported worldwide, the treatment for EHCC includes resection and molecularly targeted therapy, such as Sorafenib or Lenvatinib.^2,3,5,7–11,13–18^ 10/14 cases received only surgical resection,^2,3,7,9–11,13–16^ and among the remaining, 1 case received Sorafenib after surgical resection,^8^ 1 case received surgical resection, followed by Lenvatinib,^17^ 1 case received Sorafenib alone,^5^ and 1 did not undergo any further treatment.^18^ However, no standardized protocol and guidelines are yet available for the treatment of EHCC because of the limited number of cases and studies. Vining et al.^2^ proposed that surgical excision is a definitive treatment if technically feasible when EL gives rise to HCC. Thus, many patients without distant metastases underwent surgical resection and presented an excellent prognosis. Hitherto, no cases of EHCC with distant metastasis have been reported, and hence, no treatment has been described. Given the poor prognosis of EHCC, an optimal treatment strategy is essential. Li et al.^13^ stated that most clinicians who treat cases of EHCC follow the same guidelines applied for HCC in the mother liver. Chen et al.^6^ demonstrated that patients with early HCC are candidates for curative therapies, such as transplantation, resection, and ablation, and TACE is recommended as the first-line therapy for patients with intermediate-stage HCC, according to the European and American guidelines for the management of HCC. Therefore, we attempted to treat our case with TACE. After two TACE treatments, the patient’s condition did not deteriorate as rapidly as in the previous three months. Also, the lesions were stable, except for the increase and enlargement of intrahepatic metastases. The left sub-diaphragmatic and retroperitoneal tumors did not progress significantly. To the best of our knowledge, this is the first case report that describes TACE for EHCC treatment. Hence, we cannot make a reasonable recommendation to use TACE as the first-line therapy for patients with intermediate-stage EHCC; more cases need to be evaluated in the future.

## Conclusions

According to this review, multiple metastases in the mother’s liver and lungs of EHCC are extremely rare. The majority of the patients do not have cirrhosis or common risk factors for HCC development. Moreover, it is difficult to confirm the diagnosis of EHCC preoperatively because of the location of the mass and the rarity of the condition; thus, the diagnosis must rely on pathology. No standardized protocol and guidelines are yet available for the treatment of EHCC. If patients have surgical conditions, active surgical treatment is required. However, the effective treatment for unresectable EHCC is yet to be elucidated. Therefore, additional cases should be assimilated to determine the effect of TACE on unresectable EHCC.

## Abbreviations

EHCC: Ectopic hepatocellular carcinoma
HCC: Hepatocellular carcinoma
EL: Ectopic liver
CECT: Contrast-enhanced computed tomography
DSA: Digital subtraction angiography
IHC: Immunohistochemistry
TACE: Transarterial chemoembolization
IVC: Inferior vena cava
HBV: Hepatitis B virus
HCV: Hepatitis C virus
AFP: Alpha-fetoprotein
CA19–9: Carbohydrate antigen 19–9
CEA: Carcinoembryonic antigen

## Data Availability

The data involved in the article can be obtained through the corresponding author under reasonable conditions.
